# A Decade of Change in Sample Size and Study Setting in Leading Anesthesiology Journals: A Cross-Sectional Meta-Research Study

**DOI:** 10.7759/cureus.91767

**Published:** 2025-09-07

**Authors:** Ryoya Tajima, Yoshitaka Aoki, Yusuke Mizobuchi, Ryo Imai, Yuji Suzuki, Tetsuro Kimura, Soichiro Mimuro, Yoshiki Nakajima

**Affiliations:** 1 Department of Anesthesiology and Intensive Care Medicine, Hamamatsu University School of Medicine, Hamamatsu, JPN

**Keywords:** anesthesiology, cohort studies, publications, randomized controlled trials, sample size

## Abstract

Background

Sample size and study setting are central to the reliability and generalizability of clinical research findings. Clarifying how these design characteristics have changed over time in randomized controlled trials (RCTs) and cohort studies is essential for aligning future anesthesiology research with evolving methodological standards.

Methods

We reviewed original research articles published in 2014 and 2024 in three leading anesthesiology journals: the British Journal of Anaesthesia (BJA), Anesthesiology, and Anesthesia & Analgesia (A&A). Eligible studies included RCTs and comparative cohort studies of adult patients reporting an effect estimate with a 95% confidence interval. Sample size and the number of participating institutions were extracted. Study setting was classified as single-center or multicenter for RCTs and as single-center, multicenter, or database study for cohort studies.

Results

Among 415 screened articles, 320 met the inclusion criteria (190 from 2014 and 130 from 2024). The overall median sample size increased significantly from 132.5 (interquartile range {IQR}, 60-720) in 2014 to 1,950.5 (IQR, 133-38,338) in 2024 (P < 0.001). Median sample size increased from 80 to 120 in RCTs (P = 0.013) and from 458 to 6,617 in cohort studies (P < 0.001). The proportion of single-center RCTs did not change significantly (P = 0.55), whereas cohort studies showed a significant decrease in single-center settings (from 70.8% to 46.9%) and an increase in database studies (from 15.1% to 34.7%) (P = 0.001).

Conclusions

Studies published in 2024 showed substantially larger sample sizes than those in 2014, with cohort studies showing a marked shift toward database studies. These trends may inform the design and planning of future high-impact research in anesthesiology.

## Introduction

Sample size and study setting are critical determinants of a clinical study’s reliability and generalizability [1-3]. Larger sample sizes enhance statistical power and reduce uncertainty, while multicenter designs help ensure that findings are applicable across diverse patient populations and healthcare settings [4,5].

A meta-epidemiological analysis demonstrated that single-center randomized controlled trials (RCTs) tend to yield larger treatment effects than multicenter RCTs [4]. In observational research, the expansion of nationwide clinical registries and the increasing availability of electronic health records over the past decade have facilitated the inclusion of tens of thousands of patients in large-scale analyses [6]. These data infrastructures have enabled the examination of rare perioperative events, such as epidural hematoma, and supported multi-institutional comparisons of clinical practices [7,8]. Reflecting these methodological and infrastructural developments, the field has shown a marked shift toward large-scale, multicenter, and data-driven study designs [9,10]. This evolution has been further reinforced by journal editors and peer reviewers, who increasingly emphasize the importance of adequate sample sizes and generalizable study settings in published research [11].

Despite these developments, how the design characteristics of comparative anesthesiology studies, specifically sample size and study setting, have evolved over time remains insufficiently characterized. A particularly important question is whether the increasing use of multicenter RCTs and large-scale clinical databases has translated into substantive changes in study design. Clarifying these trends may help researchers align with current methodological expectations and guide future investigations in the field.

To address this gap, we aimed to assess temporal changes in sample size and study setting in comparative anesthesiology research. We hypothesized that recent studies are more likely to involve larger sample sizes and adopt multicenter or database-based designs compared to studies published a decade earlier. To test this hypothesis, we conducted a cross-sectional meta-research analysis of original articles published in three leading anesthesiology journals in 2014 and 2024.

## Materials and methods

Study design

This study is a cross-sectional meta-research analysis of original research articles published in three leading international anesthesiology journals: the British Journal of Anaesthesia (BJA), Anesthesiology, and Anesthesia & Analgesia (A&A). These three journals have consistently been recognized as the most influential general anesthesiology journals both in Japan and internationally [12]. As this investigation involved only publicly available information from published literature and did not include any human participants or patient-level data, approval from an institutional review board was not required.

Study selection

We selected two target years, 2014 and 2024, for analysis. Using PubMed, we searched for original research articles published in the three target journals (BJA, Anesthesiology, and A&A) in either 2014 or 2024. The search was filtered by journal name and publication year, and the final search was conducted in April 2025. Articles were eligible for inclusion if they were randomized controlled trials (RCTs) or cohort studies comparing two or more groups of adult patients based on different exposures or treatments. For cohort studies with unclear study design classification, we included them if they reported an effect estimate with a 95% confidence interval, indicating a comparative analysis. We excluded descriptive cohort studies, case series, survey-based studies, meta-analyses, review articles, letters, simulation studies, trial protocols, post hoc analyses of RCTs, studies involving pediatric populations, and studies that included the same patients multiple times for modeling or machine learning purposes. RCTs were identified using a Cochrane-recommended RCT filter [13], and cohort studies were retrieved using the Medical Subject Headings (MeSH) term “Cohort Studies” [14]. All retrieved articles were manually screened in full text by two investigators (RT and YA), who independently assessed eligibility based on predefined criteria. Discrepancies were resolved through discussion and consensus.

Outcomes

The primary outcome was the sample size, defined as the total number of patients included in the analysis of each eligible study. For RCTs, this corresponded to the number of enrolled participants. For cohort studies, the sample size referred to the number of patients included in the final analysis, which in database studies typically reflected the entire eligible population extracted from the database according to each study’s inclusion criteria. As a secondary outcome, the study setting was classified by the number of participating institutions. RCTs were categorized as single-center or multicenter and cohort studies as single-center, multicenter, or database studies. Database studies were defined as those using large-scale, pre-existing data sources such as national inpatient databases, multicenter registries, or administrative claim data. These databases generally include data from multiple institutions, although some, including the Medical Information Mart for Intensive Care (MIMIC), are derived from a single center. Studies in which multiple institutions newly collaborated for data collection, without using a pre-existing database, were classified as multicenter cohort studies.

Data collection

We extracted the following information from all eligible articles published in 2014 and 2024 in three leading anesthesiology journals (BJA, Anesthesiology, and A&A): journal name, study design (RCTs or cohort studies), sample size, and the number of participating institutions. One investigator (YA) conducted the initial data extraction, and the other (RT) verified all entries. Discrepancies were resolved through discussion.

Statistical analyses

We summarized and compared the study characteristics (i.e., journal and study design) of the articles published in 2014 and 2024. Categorical variables were presented as numbers (%) and compared using the chi-square test.

For the primary outcome (sample size), we extracted the total number of patients analyzed in each study. Descriptive statistics, including the minimum, maximum, median, and interquartile range (IQR), were calculated for studies published in 2014 and 2024. These values were summarized overall and separately by study design (RCTs and cohort studies). The Wilcoxon rank-sum test was used to compare median sample sizes between 2014 and 2024 for the overall cohort, as well as within each study design stratum. To visualize the distribution of sample sizes, overall box plots were generated. Due to the highly skewed distribution of the data, the y-axis of the box plots was presented on a base-10 logarithmic scale (log_10_) to enhance interpretability. All statistical comparisons were conducted using the original (non-log-transformed) data.

As a subgroup analysis, sample sizes in 2014 and 2024 were summarized by journal and study design. For each subgroup, box plots with base-10 logarithmic y-axes were generated to illustrate the distributions, and the Wilcoxon rank-sum test was used to compare median sample sizes between years.

For the secondary outcome (study setting), we summarized the categories as numbers (%) and compared values between 2014 and 2024 using the chi-square test. These analyses were also stratified by study design.

A two-sided p value of <0.05 was considered statistically significant. All statistical analyses were performed using Stata/MP 18 (StataCorp LLC, College Station, TX).

## Results

Selection of included studies

**Figure 1:**
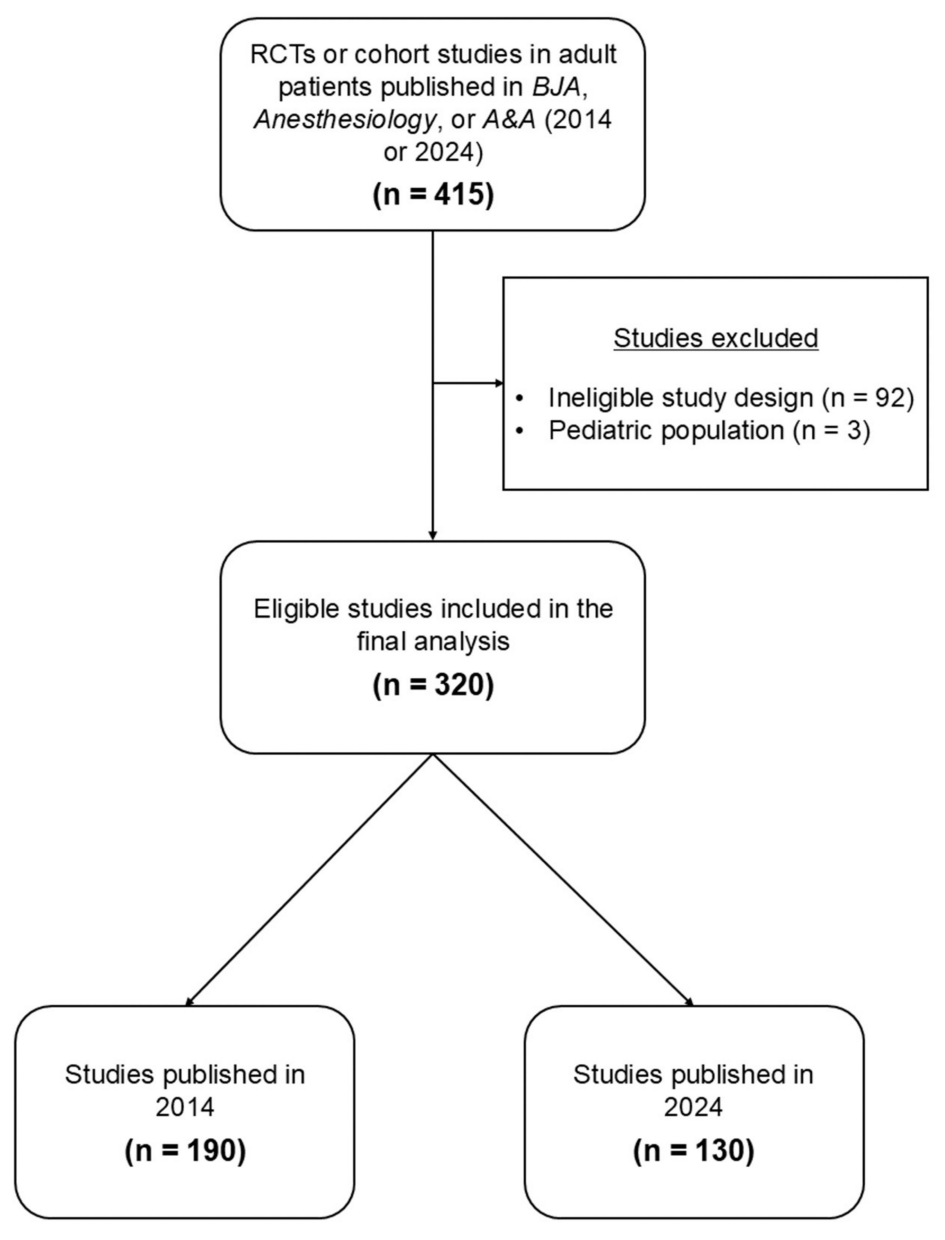
Flow diagram of study selection RCTs, randomized controlled trials; BJA, British Journal of Anaesthesia; A&A, Anesthesia & Analgesia

Figure 1 summarizes the study selection process. A total of 415 original research articles published in BJA, Anesthesiology, or A&A in either 2014 or 2024 were initially screened. After applying the exclusion criteria related to study design or patient population, 320 studies were included in the final analysis. Of these, 190 were published in 2014 and 130 in 2024.

The characteristics of the included studies by publication year are summarized in Table 1. There was no statistically significant difference in the distribution of journals between 2014 and 2024 (P = 0.06). Study design shifted over time, with the proportion of RCTs decreasing from 44.2% in 2014 to 24.6% in 2024, while that of cohort studies increased from 55.8% to 75.4% (P < 0.001).

**Table 1:** Study characteristics by publication year χ² values and corresponding p values were calculated using Pearson’s chi-square tests. Data are presented as numbers (%) RCTs: randomized controlled trials

	2014 (n = 190)	2024 (n = 130)	χ² value	P value
Journal				
British Journal of Anaesthesia	84 (44.2)	41 (31.5)	5.58	0.06
Anesthesiology	45 (23.7)	34 (26.2)		
Anesthesia & Analgesia	61 (32.1)	55 (42.3)		
Study design				
RCTs	84 (44.2)	32 (24.6)	12.82	<0.001
Cohort studies	106 (55.8)	98 (75.4)		

Sample size

**Figure 2:**
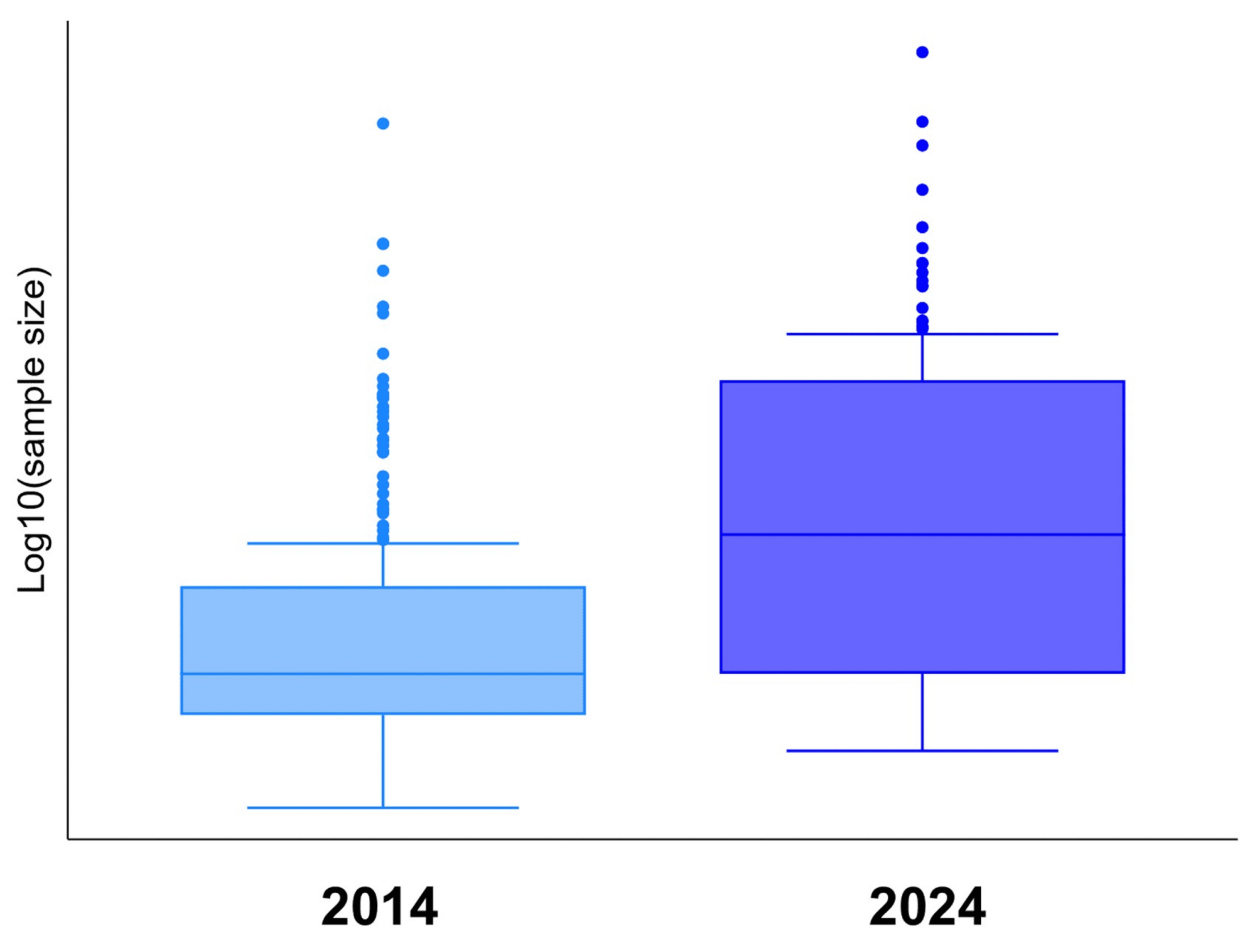
Distribution of sample sizes in 2014 and 2024 (log10 scale) Box plots showing the distribution of sample sizes in studies published in 2014 (light blue) and 2024 (blue). The y-axis is presented on a base-10 logarithmic scale (log_10_) to account for the highly skewed distribution of the data

**Figure 3:**
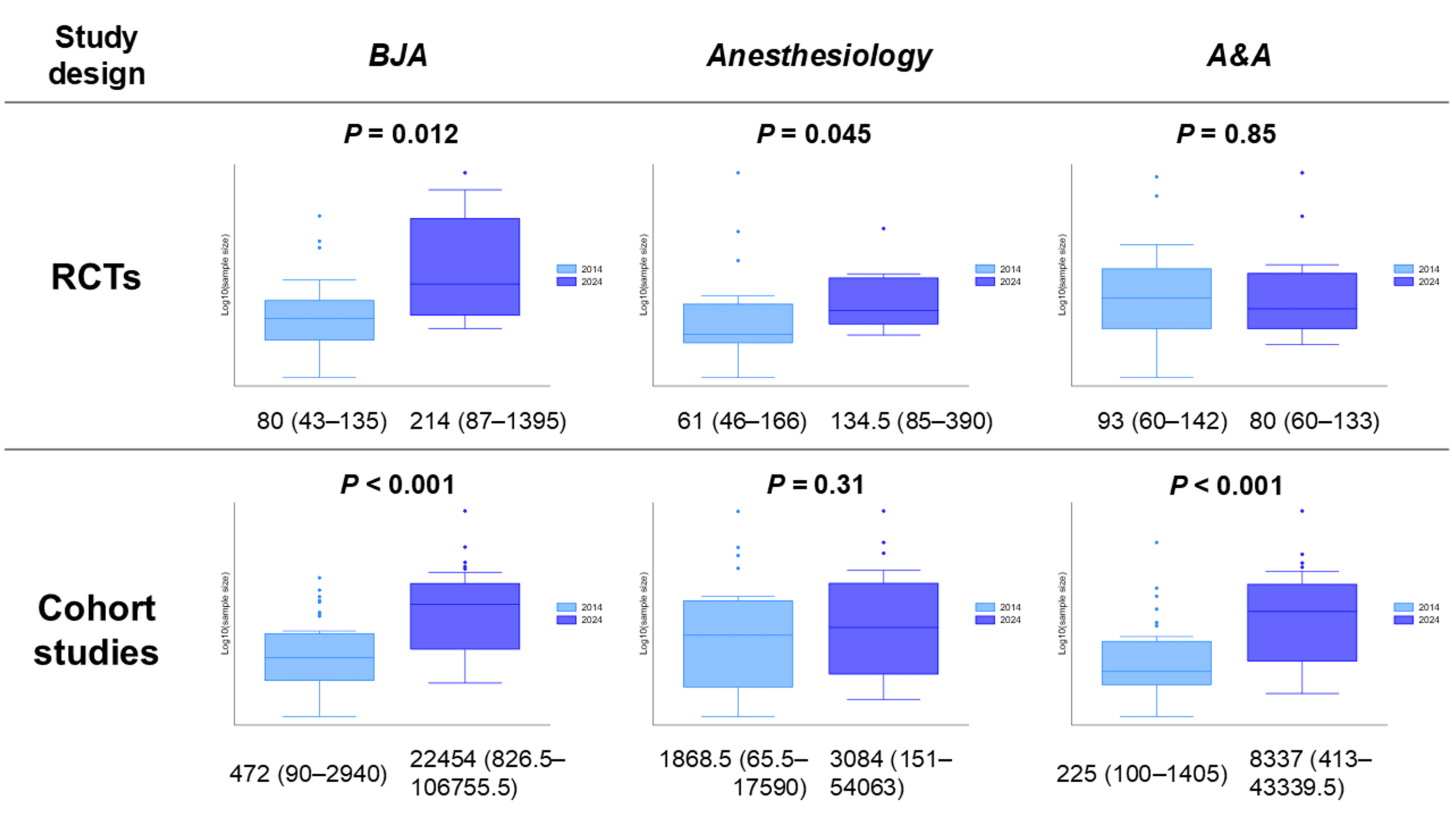
Subgroup analysis of sample size by journal and study design (log10 scale) Box plots compare studies published in 2014 (light blue) to 2024 (blue) for each journal and study design. Sample sizes on the y-axis are presented on a base-10 logarithmic scale (log_10_) to account for skewed distributions. Values shown below each plot represent the median and interquartile range. P values were calculated using the Wilcoxon rank-sum test BJA, British Journal of Anaesthesia; A&A, Anesthesia & Analgesia; RCTs, randomized controlled trials

Table 2 summarizes the sample sizes in 2014 and 2024, including the overall cohort and stratified results for RCTs and cohort studies. The overall median sample size increased significantly from 132.5 (IQR, 60-720) in 2014 to 1,950.5 (IQR, 133-38,338) in 2024 (P < 0.001). Among RCTs, the median sample size increased from 80 (IQR, 46-146) to 120 (IQR, 73.5-319) (P = 0.013). A significant increase was also observed in cohort studies, from 458 (IQR, 97-3,184) to 6,617 (IQR, 750-49,300) (P < 0.001). Figure 2 presents the distribution of sample sizes on a base-10 logarithmic scale (log_10_), visually supporting the differences between the two years. The subgroup analysis by journal and study design is shown in Figure 3. In four of the six subgroups, the median sample size in 2024 was significantly greater than that in 2014.

**Table 2:** Sample size by study design in 2014 and 2024 Z values and p values were calculated using Wilcoxon rank-sum tests for comparisons of median sample sizes IQR, interquartile range; RCTs, randomized controlled trials

	2014	2024	Z value	P value
Overall	n = 190	n = 130		
Minimum	10	30	-	-
Maximum	5,419,742	21,460,147	-	-
Median (IQR)	132.5 (60-720)	1,950.5 (133-38,338)	-6.82	<0.001
RCTs	n = 84	n = 32		
Minimum	15	48	-	-
Maximum	12,032	5,071	-	-
Median (IQR)	80 (46-146)	120 (73.5-319)	-2.49	0.013
Cohort studies	n = 106	n = 98		
Minimum	10	30	-	-
Maximum	5,419,742	21,460,147	-	-
Median (IQR)	458 (97-3,184)	6,617 (750-49,300)	-5.40	<0.001

Study setting

Study settings are summarized in Table 3, including the overall cohort and stratified results for RCTs and cohort studies. Overall, the study setting changed significantly between 2014 and 2024 (P < 0.001), with a decrease in single-center studies (77.4%-55.4%) and an increase in database studies (8.4%-26.2%). Among RCTs, no significant change in setting was observed (P = 0.55). In contrast, cohort studies exhibited a significant decrease in single-center studies from 70.8% to 46.9%, along with an increase in database studies from 15.1% to 34.7% (P = 0.001).

**Table 3:** Study setting by study design in 2014 and 2024 χ² values and p values were calculated using Pearson’s chi-square tests. Data are presented as numbers (%) RCTs: randomized controlled trials

	2014	2024	χ² value	P value
Overall	n = 190	n = 130		
Single-center	147 (77.4)	72 (55.4)	21.86	<0.001
Multicenter	27 (14.2)	24 (18.5)		
Database	16 (8.4)	34 (26.2)		
RCTs	n = 84	n = 32		
Single-center	72 (85.7)	26 (81.3)	0.35	0.55
Multicenter	12 (14.3)	6 (18.8)		
Cohort studies	n = 106	n = 98		
Single-center	75 (70.8)	46 (46.9)	13.41	0.001
Multicenter	15 (14.2)	18 (18.4)		
Database	16 (15.1)	34 (34.7)		

## Discussion

This study confirmed our hypothesis that comparative anesthesiology research has undergone measurable changes in recent years. Sample sizes increased significantly between studies published in 2014 and 2024, with a particularly pronounced rise in cohort studies. Similar patterns were observed in subgroup analyses stratified by journal and study design, supporting the robustness of our findings. While the proportion of single-center studies remained unchanged among RCTs, cohort studies showed a clear shift toward database-based designs.

A key finding of this study is that sample sizes have significantly increased over time in both RCTs and cohort studies. Chow et al. compared RCTs published in six leading anesthesiology journals in 2010 and 2016, reporting a median sample size increase from 63 (IQR, 41-101) to 80 (IQR, 52-136) [11]. This is comparable to the modest increase observed in the present study’s RCTs, from 80 to 120 participants. In our study, we observed a more pronounced increase in sample size among cohort studies, from 458 to 6,617, a novel finding, as previous reports have primarily focused on RCTs. This discrepancy may reflect greater availability and the use of large-scale data sources in observational research, as well as the improved feasibility of multicenter retrospective analyses [8,15,16]. The large sample sizes observed in 2024 studies are essential for enhancing statistical power, reducing uncertainty, and enabling the analysis of rare perioperative outcomes.

Our results also indicate a substantial shift toward database study in comparative cohort studies. Between 2014 and 2024, the proportion of single-center cohort studies decreased, while database analyses more than doubled, rising from 15.1% to 34.7%. This shift may be attributed to improved access to clinical registries, administrative datasets, and electronic health records, which allow investigators to analyze data from large, diverse patient populations with minimal additional data collection burden [17,18]. However, database studies also introduce methodological challenges, including data heterogeneity, limited variable granularity, and potential unmeasured confounding, all of which must be addressed through rigorous study design and analytic methods.

Our findings have important implications for how anesthesiology research is designed, conducted, and interpreted. First, to our knowledge, this is the first study to demonstrate a substantial increase in the sample sizes observed among studies published in the three leading anesthesiology journals over the past decade. These benchmarks for RCTs and cohort studies may help guide researchers aiming to publish in high-impact journals. Second, while single-center RCTs continue to be accepted, the proportion of single-center cohort studies has declined markedly from 2014 to 2024. This trend highlights the value of designing observational studies with a broader scope and infrastructure. Third, we observed a relative decline in published RCTs and a concurrent increase in cohort studies. This shift may reflect the increasing difficulty of conducting RCTs due to financial and logistical barriers, along with the fact that large-scale multicenter RCTs in anesthesiology are now published in high-impact general medical journals such as The New England Journal of Medicine and The Lancet [19,20]. In this context, registry-based cohort research has offered a practical and impactful alternative. Our group has previously published multicenter cohort studies using the Japanese Intensive care PAtient Database (JIPAD), including articles accepted by BJA and A&A, highlighting the potential of nationwide clinical data infrastructure [21-27]. Building on this model, the establishment of a dedicated research database by the Japanese Society of Anesthesiologists may help expand clinical research capacity and elevate the international presence of Japanese anesthesiologists [28].

This study has several limitations. First, we compared studies from only two discrete time points, 2014 and 2024, without examining the intervening years. Although this design allowed for clear cross-sectional comparisons, it precludes the evaluation of longitudinal trends or gradual shifts in research practices. Second, we focused solely on quantitative aspects of the study scale, namely, sample size and the number of participating institutions. We did not evaluate study content, clinical domains, outcomes assessed, or methodological rigor, which may also have evolved over time and influenced the overall quality and impact of published research [29]. Third, our analysis was limited to three leading general anesthesiology journals (Anesthesiology, BJA, and A&A). As a result, studies published in subspecialty or regional journals were not included, which may limit the generalizability of our findings to the entire field of anesthesiology. Fourth, a number of cohort studies published in 2014 lacked clear methodological descriptions, making it difficult to accurately determine inclusion eligibility, sample sizes, or the number of centers. Although two reviewers independently screened and extracted data using systematic review principles, the potential for misclassification or underreporting remains. Fifth, two of the six subgroup analyses did not reach statistical significance, which limits the certainty of subgroup-level inferences; however, the absence of any significant decrease supports the robustness of the overall conclusion that sample sizes have increased. Finally, the present study included RCTs and comparative cohort studies while excluding other types of research, such as descriptive studies, modeling papers, and studies employing machine learning techniques. This restriction was necessary to ensure conceptual comparability with RCTs and to minimize heterogeneity introduced by emerging study designs that differ substantially in methodology and purpose [30].

## Conclusions

This study demonstrates that comparative anesthesiology research has undergone substantial changes in design and scale over the past decade. Sample sizes have increased considerably, particularly in cohort studies, reflecting broader access to clinical data and growing expectations for statistical power and generalizability. The use of database-based designs has become more prevalent, while single-center cohort studies have declined, suggesting a shift toward multi-institutional research infrastructure. Meanwhile, the relative decrease in RCTs and increase in cohort studies may reflect evolving methodological preferences and practical considerations in the field. Taken together, these findings provide contemporary benchmarks for sample size and study setting that can inform the design, evaluation, and planning of future investigations in anesthesiology.

## References

[1] Ranganathan P, Deo V, Pramesh CS (2024). Sample size calculation in clinical research. Perspect Clin Res.

[2] Hobart JC, Cano SJ, Warner TT, Thompson AJ (2012). What sample sizes for reliability and validity studies in neurology?. J Neurol.

[3] Althubaiti A (2023). Sample size determination: a practical guide for health researchers. J Gen Fam Med.

[4] Dechartres A, Boutron I, Trinquart L, Charles P, Ravaud P (2011). Single-center trials show larger treatment effects than multicenter trials: evidence from a meta-epidemiologic study. Ann Intern Med.

[5] Das MK (2022). Multicenter studies: relevance, design and implementation. Indian Pediatr.

[6] Abdel-Kader AK, Eisenkraft JB, Katz DJ (2021). Overview and limitations of database research in anesthesiology: a narrative review. Anesth Analg.

[7] Liem VG, Hoeks SE, van Lier F, de Graaff JC (2018). What we can learn from big data about factors influencing perioperative outcome. Curr Opin Anaesthesiol.

[8] Dutton RP (2015). Large databases in anaesthesiology. Curr Opin Anaesthesiol.

[9] Boet S, Burns JK, Cheng-Boivin O (2021). Mapping multicenter randomized controlled trials in anesthesiology: a scoping review. Syst Rev.

[10] Pando A, Tenneli AK, Pradeep T, Augustine P, Krishna B, Raj JP (2025). Acute pulmonary edema after subarachnoid hemorrhage: risk factors and comorbidities-an analysis of a nationwide database from the United States. J Intensive Care.

[11] Chow JT, Turkstra TP, Yim E, Jones PM (2018). Sample size calculations for randomized clinical trials published in anesthesiology journals: a comparison of 2010 versus 2016. Can J Anaesth.

[12] Hirota K (2013). A worrying decline in anesthesia journal publications from Japan. J Anesth.

[13] Glanville J, Kotas E, Featherstone R, Dooley G (2020). Which are the most sensitive search filters to identify randomized controlled trials in MEDLINE?. J Med Libr Assoc.

[14] Fatehi F, Bird D, Gray LC (2013). PubMed searching using MeSH terms to identify randomized controlled trials on telemedicine for diabetes. J Telemed Telecare.

[15] Chau HD, Gan ZJ, Bin Abd Razak HR, Allen JC, Koh SJ, Howe TS (2024). A review of authorship inflation and multicenter collaboration trends in orthopedic, medical, and surgical journals over the last 60 years. Cureus.

[16] Delange B, Popoff B, Séité T, Lamer A, Parrot A (2025). LinkR: an open source, low-code and collaborative data science platform for healthcare data analysis and visualization. Int J Med Inform.

[17] Yasunaga H (2024). Updated information on the diagnosis procedure combination data. Ann Clin Epidemiol.

[18] Yajima W, Aso S, Matsui H, Fushimi K, Yasunaga H (2025). Association between initial intravenous fluid volume and the composite outcome of hemodialysis dependence at discharge or in-hospital mortality in inpatients with rhabdomyolysis. J Intensive Care.

[19] Neuman MD, Feng R, Carson JL (2021). Spinal anesthesia or general anesthesia for hip surgery in older adults. N Engl J Med.

[20] Sessler DI, Pei L, Li K (2022). Aggressive intraoperative warming versus routine thermal management during non-cardiac surgery (PROTECT): a multicentre, parallel group, superiority trial. Lancet.

[21] Omoto M, Aoki Y, Nakajima M (2025). Epidemiological investigation of unplanned intensive care unit admissions from the operating room after elective surgery: a nationwide observational study in Japan. Anesth Analg.

[22] Aoki Y, Nakajima M, Mimuro S (2025). Association between postoperative body temperature and in-hospital mortality: a nationwide cohort study of 157,028 critically ill patients in Japan. Can J Anaesth.

[23] Aoki Y, Nakajima M, Sugimura S (2023). Postoperative norepinephrine versus dopamine in patients undergoing noncardiac surgery: a propensity-matched analysis using a nationwide intensive care database. Korean J Anesthesiol.

[24] Asaba H, Aoki Y, Akinaga C (2023). Obstetric admission to intensive care units in Japan: a cohort study using the Japanese Intensive care PAtient Database. J Anesth.

[25] Niwa T, Aoki Y, Kobayashi K, Suzuki K, Katsuragawa T, Mimuro S, Nakajima Y (2025). Effect of pulmonary artery catheters in patients with cardiovascular diseases stratified based on severity: a propensity score-matched analysis. J Anesth.

[26] Suzuki Y, Aoki Y, Shimizu M (2024). Predictive accuracy of lactate albumin ratio for mortality in intensive care units: a nationwide cohort study. BMJ Open.

[27] Anezaki H, Aoki Y, Kato H (2025). Perioperative cardiac arrest requiring admission to intensive care units in Japan: epidemiological differences between emergency and elective surgery. Br J Anaesth.

[28] Saito J, Hirota K, Mazda Y (2024). Fixing the anesthesia research crisis in Japan. J Anesth.

[29] Granholm A, Anthon CT, Kjær MN (2022). Patient-important outcomes other than mortality in contemporary ICU trials: a scoping review. Crit Care Med.

[30] Toews I, Anglemyer A, Nyirenda JL (2024). Healthcare outcomes assessed with observational study designs compared with those assessed in randomized trials: a meta-epidemiological study. Cochrane Database Syst Rev.

